# 54 Prophylactic Antibiotics are Unnecessary for Routine CO2 Laser Burn Scar Treatment

**DOI:** 10.1093/jbcr/irad045.028

**Published:** 2023-05-15

**Authors:** Records Kevin, Brittany Munnerlyn, Steven Kahn

**Affiliations:** MUSC, Charleston, South Carolina; MUSC, Charleston, South Carolina; The South Carolina Burn Center/Medical University of South Carolina, Charleston, South Carolina; MUSC, Charleston, South Carolina; MUSC, Charleston, South Carolina; The South Carolina Burn Center/Medical University of South Carolina, Charleston, South Carolina; MUSC, Charleston, South Carolina; MUSC, Charleston, South Carolina; The South Carolina Burn Center/Medical University of South Carolina, Charleston, South Carolina

## Abstract

**Introduction:**

Many providers utilize prophylactic cephalexin prior to Co2 ablation laser for hypertrophic scarring. Many providers use this as standard of treatment despite a paucity of high-quality literature supporting the practice. The purpose of this study is to report a single-center experience in performing laser-assisted delivery of triamcinolone and destruction of burn scars without the use of antibiotics.

**Methods:**

This was a retrospective descriptive study from a single center with a new laser scar program. The first 14 patients received cephalexin 500mg by mouth every 6 hours starting the day of the procedure for 5 days and valaciclovir 1000mg by mouth daily for 5 days for their first two procedures- as per manufacturer recommendations. After that, cephalexin was no longer utilized nor were any antibiotics given at the start time of procedure. Valacyclovir is given on a case-by-case basis for patients with a history of cold sores, known herpes simplex, or extensive facial procedures. Burn team staff members followed up with the patients within 48 -72 hours by phone call. Burn staff called patients after each laser treatment to assess for any adverse reactions. Adverse reactions include open areas, rashes, or blisters to laser site. The two groups were compared with a Fisher’s exact test for number of infections.

**Results:**

Patients who received cephalexin accounted for the first 28 cases and none developed an infection. In 201 cases without antibiotics, only one patient developed cellulitis (0.5%), p=1. This patient was noncompliant with restrictions on hand hygiene and used a cheap lotion with parabens, dyes, and perfumes on post operative day 2. Another patient experienced a self-limiting dermatitis on their back of uncertain etiology. In 36 cases, valacyclovir was given. None of the patients in either group developed vesicular lesions consistent with HSV.

**Conclusions:**

There were no significant differences in number of infections whether patients treated with or without prophylactic antibiotics before laser treatment of hypertrophic burn scars. The authors continue to treat patients without administering prophylactic antibiotics.

**Applicability of Research to Practice:**

laser programs are common in burn centers

